# Case Report of Extended Survival and Quality of Life in a Melanoma Patient with Multiple Brain Metastases and Review of Literature

**DOI:** 10.7759/cureus.1947

**Published:** 2017-12-14

**Authors:** William Sperduto, David M King, Yoichi Watanabe, Emil Lou, Paul W Sperduto

**Affiliations:** 1 Department of Medicine, Division of Hematology, Oncology and Transplantation, University of Minnesota; 2 Medical Oncology, University of Minnesota; 3 Radiation Oncology, University of Minnesota; 4 Minneapolis Radiation Oncology & Gamma Knife Center, University of Minnesota

**Keywords:** melanoma, brain metastases, outcomes

## Abstract

Long-term survival for melanoma patients with multiple brain metastases is rare. A review of the literature reveals only three reported melanoma patients with multiple brain metastases who survived more than 10 years. We present a patient who is recurrence-free 11 years after the diagnosis of three brain metastases. Her treatment consisted of cytokine (interferon and interleukin-2) and chemotherapy nine months prior to developing brain and soft tissue metastases, which were treated with stereotactic radiosurgery and stereotactic ablative radiotherapy, respectively, followed by six months of chemotherapy. Notably, she has not received any treatment for over 10 years, never underwent craniotomy or whole brain radiation therapy, currently has a perfect score on the functional assessment of cancer therapy for brain (FACT-Br) quality of life (QoL) scale, and runs marathons. This treatment course is consistent with emerging literature on the abscopal effect (radiation-induced immune response). Clinical trials are needed to better understand and harness the abscopal effect in order to optimally integrate targeted drug and radiation therapies.

## Introduction

Brain metastases are a common and complicated oncologic problem. Approximately 120,000-170,000 patients are diagnosed each year with brain metastases in the United States and an estimated 20% of all patients who die from cancer will develop brain metastases [1]. Of all malignancies, melanoma has the highest propensity to metastasize to the brain [1]. Melanoma is also of intense research interest because it is sensitive to immune modulation and radiation-induced immune response (the abscopal effect) [2-3]. The abscopal effect is a phenomenon in which local radiotherapy is associated with the regression of other nonirradiated tumors. This phenomenon was rare in the past but is more frequently reported since technological advances have made feasible the precise delivery of high doses of radiation [stereotactic radiosurgery (SRS) and stereotactic ablative radiotherapy (SABR)]. In this setting, the radiation may act as an in situ vaccine [4].

Clinically, quality of life (QoL) is often more important to these patients than longevity; QoL is a key measurement of survivorship for all cancer patients. Several methods to measure patient QoL are available. One such method is the functional assessment of cancer therapy for brain (FACT-Br)—a widely accepted, commonly employed, patient-reported, statistically validated QoL tool for patients with brain tumors [5]. This questionnaire offers important insight into both the patient’s QoL and the impact of cancer treatment on QoL. The FACT-Br consists of 50 questions covering multi-item domains, such as physical, social/family, emotional, and functional well-being, as well as 23 questions regarding brain-specific QoL concerns.

The purpose of this report is to present a remarkable melanoma patient with multiple brain metastases, include her QoL, and review the medical literature on long-term survivors of melanoma with multiple brain metastases.

## Case presentation

A 36-year-old, white, female, amateur marathon runner presented in August 2005 with a right neck mass. Fine needle aspiration initially confirmed a malignancy, which was later confirmed as a malignant melanoma by excisional biopsy of a posterior scalp lesion on 9-15-05. This malignant melanoma was histopathologically staged as Clark’s Level IV, with a Breslow depth of at least 6 mm, angiolymphatic invasion, and positive, deep and peripheral margins. Brain magnetic resonance imaging (MRI) for initial radiologic staging on 9-27-05 showed multiple scalp lesions but no evidence of parenchymal brain metastases. A positron emission tomography (PET) scan on 9-27-05 showed hypermetabolic activity only in the left neck. On 10-11-05, she underwent a left, modified radical neck dissection and a wide local excision of the scalp lesion. Pathology confirmed metastatic melanoma in three of 28 lymph nodes with extension into the adjacent soft tissues in two areas. Pathology from the scalp excision showed a maximum tumor depth of 1.9 cm and the deep margin remained positive. She underwent two additional scalp excisions and the deep margin remained positive. Her stage was T4bN2bM0, stage IIIC. She received 64 Gy radiation therapy to the left neck and scalp, which was completed on 1-20-06. She then received three cycles of cisplatinum, interferon, and vinblastine followed by interleukin-2, which was completed in March 2006. She did well without evidence of recurrence until November 2006 when she underwent a debridement of necrotic tissue in the scalp lesion. A PET scan on 12-5-06 showed a 0.7 cm hypermetabolic nodule in the retroperitoneum consistent with metastatic recurrence.

**Figure 1:**
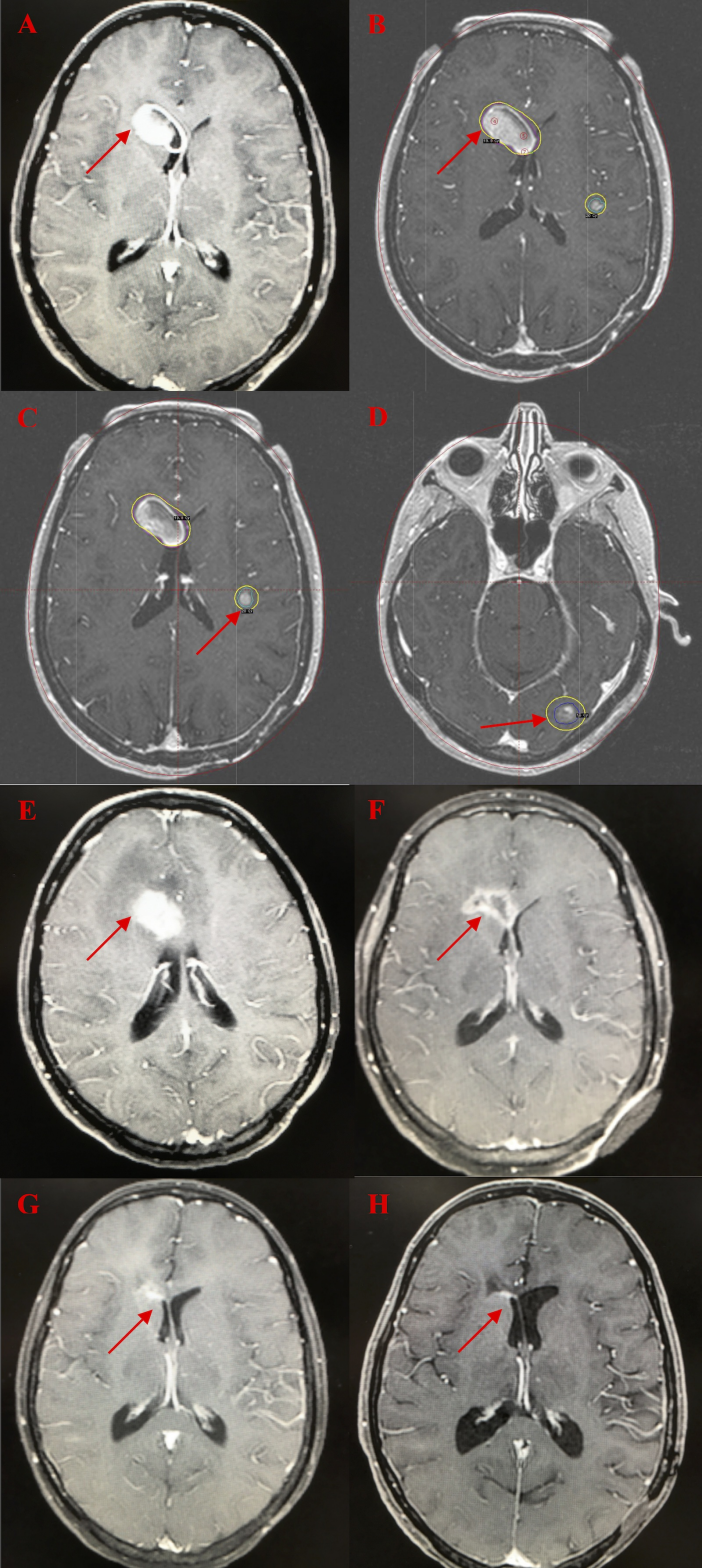
Serial Brain MRI Images 1A: Initial magnetic resonance imaging (MRI) shows the largest of three brain metastases, 12-06-2006 1B: Gamma Knife plan for right frontal brain metastasis, 12-13-2006 1C: Gamma Knife plan for left frontal brain metastasis, 12-13-2006 1D: Gamma Knife plan for left occipital brain metastasis, 12-13-2006 1E: MRI 9 months after Gamma Knife shows marked radiation necrosis and edema, 9-26-07 1F: MRI 18 months after Gamma Knife shows resolving radiation necrosis, 5-23-2008 1G: MRI 21 months after Gamma Knife shows minimal residual enhancement, 10-23-2008 1H: MRI 10.7 years after Gamma Knife shows no evidence of disease, 8-02-2017

A brain MRI on 12-6-06 showed three brain metastases (2.5 cm right caudate, 1.1 cm left parietooccipital, and 0.7 cm left posterior frontal) (Figure 1A), which were not present on the prior scan on 6/22/06. Whole brain radiation therapy was not given (and has not been given) due to the prior scalp radiation. She underwent stereotactic radiosurgery (SRS) (Gamma Knife, Elekta, Stockholm, Sweden) to all three lesions on 12-13-06: right caudate, 20 Gy to a volume of 8.4 cc (Figure 1B); left posterior frontal, 24 Gy to a volume of 0.47 cc (Figure 1C); and left parietooccipital, 24 Gy to a volume of 1.6 cc (Figure 1D). She underwent stereotactic ablative radiotherapy (SABR) to the pelvic soft tissue metastasis (25 Gy x 5 over two weeks, completed on 2/23/07). Between March and June 2007, she received four cycles of carboplatin, paclitaxel, and temozolomide treatment. In September 2007, she developed headaches, nausea, vomiting, and confusion. An MRI on 9-26-07 showed a marked increase in enhancement and edema in the right frontal lobe consistent with radiation necrosis (Figure 1E). Due to increased headaches and possible radiation necrosis, the temozolomide was discontinued. The edema was treated with steroids, which were gradually tapered off over four months. A brain MRI on 5-23-08 showed improvement with central necrosis of the previously solid-appearing lesion (Figure 1F). A brain MRI on 10-23-08 showed further resolution of the enhancement/necrosis with minimal residual enhancement (Figure 1G). Serial imaging since that time has shown no evidence of recurrent tumor or necrosis. She has received no treatment since September 2007. She remains clinically and radiographically free of disease 11 years after the diagnosis of multiple brain metastases and more than 10 years after completion of treatment. A brain MRI on 8-2-17 showed no change in the minimal residual enhancement/scar tissue (Figure 1H) and a PET scan 8-2-17 showed no evidence of disease. She has remained asymptomatic for over a decade and continues to run marathons, as recently as 10-14-17. In November 2017, she completed the FACT-Br questionnaire, a patient-reported QoL tool to reassess brain cognition. Her FACT-Br score was perfect (200 on a scale of 200), 11 years after diagnosis of her brain metastases. Notably, this patient never underwent craniotomy or whole brain radiation therapy and, consequently, avoided the related long-term neurocognitive toxicity of these interventions.

## Discussion

Historically, the diagnosis of melanoma with brain metastasis was considered uniformly fatal with survival in the two- to four-month range [1]. It is a complex problem because of the marked heterogeneity of this patient population and the wide range of prior treatments (none vs. extensive) they may have received at the time of diagnosis of the brain metastases. Because of this heterogeneity, estimating a prognosis remains challenging. A diagnosis-specific prognostic index, the graded prognostic assessment (GPA), has been published, confirming survival varies widely by diagnosis and diagnosis-specific prognostic factors [6]. These studies have shown that the median survival for patients with brain metastases is improving; however, long-term survivors are rare, and those with multiple brain metastases surviving more than 10 years are exceedingly rare.

**Figure 2:**
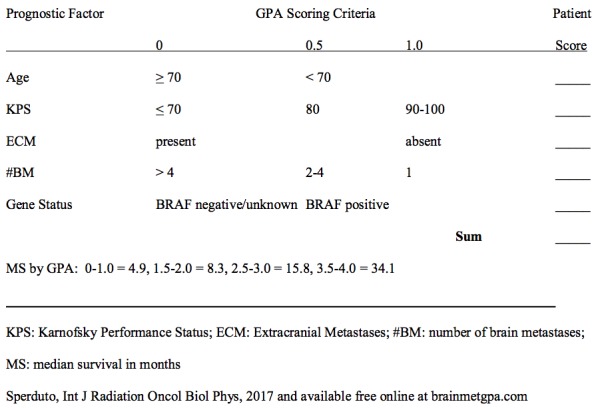
Melanoma-molGPA Worksheet melanoma-molGPA: graded prognostic assessment for melanoma using molecular markers

To fully appreciate this patient’s remarkable outcome, it is appropriate to review how her outcome compares to the best available evidence of survival for melanoma patients with brain metastases. We recently updated and published the GPA for melanoma using molecular markers or melanoma-molGPA [7] based on a multi-institutional retrospective study of 483 melanoma patients with brain metastases diagnosed between 1-1-2006 and 12-31-15. Notably, the patient presented here was diagnosed in 2006, so she is a contemporary of the patients in the melanoma-molGPA update study. The study showed five prognostic factors significant for survival. Figure 2 shows a worksheet with which to calculate the melanoma-molGPA. To further simplify the calculation of the melanoma-molGPA, a free user-friendly app is available at brainmetgpa.com. The overall median survival has improved from six to 10 months since the 1980s, and the median survival by melanoma-molGPA groups for GPAs of 0-1.0, 1.5-2.0, 2.5-3.0, and 3.5-4.0 was 4.9, 8.3, 15.8, and 34.1 months, respectively. The patient presented here had a melanoma-molGPA of 3.0 on a 4.0 scale on both the original and updated GPA index, correlating with an estimated predicted survival of 8.8 and 15.8 months, respectively. This exceptional responder is disease-free and asymptomatic with a perfect FACT-Br QoL score 11 years after the diagnosis of multiple brain metastases. 

A search of the medical literature reveals that long-term survivors (greater than 10 years) in patients with multiple brain metastases are exceedingly rare. There are only three published reports of melanoma patients with multiple brain metastases surviving longer than 10 years (Table 1).

**Table 1:** Melanoma Patients with Multiple Brain Metastases and 10-Year Survival *None of these long-term survivors received whole brain radiation therapy. SBRT: stereotactic body radiotherapy; SRS: stereotactic body radiotherapy

Author	Survival (years)	Treatment
Current Report	11	Extracranial surgery, interferon, interleukin, chemotherapy, SBRT, SRS
Weiss [8]	12	Interferon, SRS, two craniotomies, chemotherapy
Bordry [9]	13	Craniotomy, vaccine, SRS
Hamid [10]	16	SRS, two craniotomies, chemotherapy

Weiss et al. reported a 12-year survival for a patient who underwent chemotherapy and multiple craniotomies. Molecular tumor profiling and a genetic variant analysis suggested that specific germline variants and single nucleotide polymorphisms (SNPs) may activate the immune system, improve prognosis, and guide personalized treatment plans [8]. Bordry et al. presented a 13-year survivor with pelvic adenopathy and brain metastases [9]. The patient was treated in two clinical trials (NCT001112216 and then NCT00112242). That patient underwent surgery, received vaccine therapy (six cycles of MAGE-A10 and Melan-A peptides every three weeks), maintenance therapy of seven monthly subcutaneous injections of MAGE-A10, Melan-A, and NY-ESO-1, followed by monthly injections of the same peptides with adjuvant montanide and CpG 7909. After three monthly vaccinations of that regimen, the patient developed very strong subcutaneous reactions at the injection sites, representing the first case of cancer vaccine-induced granulomas in melanoma patients. Therefore, the next 33 vaccinations included only CpG as the adjuvant, without montanide. Hamid et al. published the longest reported survival for a melanoma patient with multiple brain metastases [10]. That patient, with a 16-year survival, underwent surgical resection, SRS, and chemotherapy.

A common characteristic among these rare case reports is multidisciplinary management. However, our patient is the only one who never underwent a craniotomy. Notably, neither our patient nor any of the other three 10+ year survivors of melanoma with multiple brain metastases received whole brain radiation therapy.

We postulate that the prolonged survival in our patient and the aforementioned long-term survivors may be attributed to the abscopal effect—a phenomenon in which local radiotherapy is associated with the regression of metastatic cancer at a distance from the irradiated site. Activation and even hyperstimulation of the immune system, directed against malignancies, may mediate the abscopal effect. Postow et al. published a case report showing the abscopal effect in a patient with metastatic melanoma when treated with ipilimumab and SABR (9 Gy x 3) for a paraspinal metastasis [3]. The report showed that other metastases decreased in size, and the SBRT released antigens that correlated with the abscopal effect. The features of the patient presented here suggest that the abscopal effect included: 1) she received cytokine therapy in the form of interferon and interleukin-2 (9-12 months prior to SRS and SABR); 2) she underwent SRS (20 Gy to the 50% isodose line (IDL) to each of the three brain metastases), which resulted in marked radiation necrosis and cerebral edema, requiring months of steroids but no craniotomy; 3) she underwent SABR (5 Gy x 5) to a soft tissue metastasis in the pelvis in February 2007 (before such treatment was well established), which may have released antigens contributing to the long-term control of the brain metastases; 4) the time course of the radiation necrosis, documented in Figure 1, is consistent with the “bloom and wilt” course of radiation necrosis; 5) the time course of the cytokine therapy nine to 12 months prior to SRS and SABR followed by marked inflammation/necrosis is consistent with the post-treatment antigen-release and related inflammation seen in Postow’s case report [3] and in immunotherapy clinical trials [2,4]; and 6) of all malignancies, this patient had melanoma, which has a high propensity to spread to the brain [1] and is sensitive to immunotherapies [2-4]. Notably, this patient received interferon and interleukin-2 but was treated before other immunotherapies were available. It is conceivable that both the SRS and SABR activated an immune response, resulting in antigen release, effectively an in situ immunotherapy that subsequently contributed to long-term tumor control and survival. Confirmation of the abscopal effect is an area of active research and has been documented by increased antigen levels after treatment [3], but those tests were not performed in the patient presented here. Therefore, we can only report her treatment and outcome are consistent with but not proof of an abscopal effect.

## Conclusions

The reasons for the extended survival of the patient presented and in cited literature remain unknown, but increasing evidence shows that radiation can act as an immune stimulus, recruiting immune mediators that enable anti-tumor responses within and outside (the abscopal effect) the radiation field. There has been a rapid expansion of phase I and II clinical trials using radiation therapy to enhance antitumor immunity. The potential ability of radiation to induce an immunogenic cell death and counteract an immune-suppressive tumor microenvironment for effectively converting the irradiated tumor into an in situ vaccine has implications for both local and systemic disease control and is the focus of many current clinical trials. Quality of life is considered of equal or greater importance than length of life for most cancer patients and must remain a key endpoint in clinical trials. In the future, with an improved understanding of evolving treatment options, their interaction, and prudent judgment regarding how to sequence these therapies and when to use or not use them, hopefully, there will be many more patients like the patient described here.
